# Utility of Combining a Simulation-Based Method with Lecture for Retinopathy Training in Emergency Medicine Residency

**DOI:** 10.51894/001c.137284

**Published:** 2025-04-30

**Authors:** Kevin Durell, Arlen Hooley

**Affiliations:** 1 Compliance Education Henry Ford Health; 2 Compliance Office HFPACO-Mosaic ACO; 3 Department of Medical Specialties, Center for Health Innovation and Education MSUCOM; 4 Emergency Medicine Henry Ford Jackson Health; 5 Emergency Medicine Specialist Kaiser Permanente Manteca Medical Center

**Keywords:** Simulation-Based Training, Funduscopic Examination, Emergency Medicine Residency, Retinopathy Diagnosis, Medical Education, Clinical Skill Improvement

## Abstract

**INTRODUCTION:**

Funduscopic examination is a critical skill for diagnosing eye-related pathologies but has witnessed a decline in proficiency over recent decades. Simulation-based training is proposed as a solution to enhance emergency medicine residents’ funduscopic examination skills. We hypothesized that a combination of lecture and simulation would improve residents’ diagnostic abilities, with senior residents potentially outperforming junior counterparts.

**METHODS:**

This study aimed to assess the effectiveness of simulation-based training in improving the funduscopic examination skills of emergency medicine residents and whether factors such as seniority or prior ophthalmology rotation influenced the results. Residents participated in a 10-question image-based exam, with alternating pairs viewing images and answering questions. Simulation equipment, including digital eye examination retinopathy trainers, was utilized for the study. A lecture covering possible answers was provided, followed by a second round of testing.

**RESULTS:**

A total of 20 participants in this pilot study took both the pre- and post-lecture tests. Test scores significantly improved after supplemental education, indicating the effectiveness of simulation-based training in enhancing funduscopic diagnostic skills. Interestingly, resident year and prior completion of an ophthalmology rotation did not significantly impact test scores, underscoring the importance of supplemental education. Notably, participants demonstrated high accuracy in identifying Normal Fundus and several specific pathologies post-training.

**CONCLUSION:**

Simulation-based training, supplemented by lectures, offers a promising avenue for improving funduscopic examination proficiency among emergency medicine residents. This study’s findings highlight the potential for standardized training methods to benefit residents across different levels of experience. Future research could explore the long-term retention of these skills and their translation into clinical practice. In an era where technological advancements are reshaping medical education, simulation-based training offers a promising avenue for ensuring that essential clinical skills are not lost but rather strengthened among medical professionals.

## INTRODUCTION

Funduscopic examination is a physical examination skill that uses an ophthalmoscope to allow visualization of the retina and associated structures. It can be used to diagnose and differentiate emergent and non-emergent pathologies that involve these structures. It is an integral part of any emergency medical examination of the eye.^1^ In medical school, this skill is taught with other physical examination tests as part of a comprehensive physical exam, specifically the neurological examination.^2^ However, this skill has declined over the course of several decades.^3^ Teaching the direct ophthalmoscope examination has been hindered by several factors, one of which is the inherent challenge of verifying/confirming the preceptor’s observations with the student. Also, the skill’s decline in usage and the difficulty of training has resulted in its limited application in the field of clinical medicine.^4^

Simulation training is one way to meet the need to train emergency medicine residents in multiple different physical examination and procedural skills.^5^ Per the Accreditation Council for Graduate Medical Education (ACGME) competencies and requirements, simulation is an integral part of graduate medical education.^6^ Despite this, there has been limited research on the effectiveness of simulation-based training or modalities for assessing understanding (skill learning? Comprehension?) in emergency medicine.^7,8^ A recent study published in JAMA Neurology showed the utility of a simulation-based method of funduscopic training with a lecture model in neurology residency.^9^ We hypothesized that a before and after study using a combination of lecture and simulation will increase the likelihood of correct diagnosis of funduscopic pathologies in a small cohort of emergency residents. A secondary hypothesis is that the senior residents in the cohort, residents at years 3 and 4 of training or those that have already completed an ophthalmology rotation, will outperform their junior counterparts or those that have not completed an ophthalmology rotation.

## METHODS

### Standard Protocol Approval and Informed Consent

The study protocol and informed consent were approved by the Henry Ford Allegiance Health Institutional Review Board at our hospital prior to any data collection (IRB#547). All emergency medicine residents were invited to participate in the study on January 17, 2020, on a voluntary basis, and were given a written description of study aims and structure. Written informed consent was obtained from all participants, and data de-identification was achieved using study identification numbers generated and managed in accordance with a set of study protocol elements outlined below. An independent departmental office maintained secure storage of all answer sheets and was responsible for data analysis and for protecting the anonymity of the study participants.

### Study design

We conducted a prospective, single-blind educational research study by enrolling a cohort of emergency medicine residents at a community level II trauma medical center. Of the 31 emergency medicine residents, 20 were present for the regularly scheduled didactic day. All 20 accepted the invitation to volunteer for the study. Residents would not be allowed to use electronic devices during the study, including the use of cell phones except in emergencies. To further that end and decrease boredom, a movie was played (*Spiderman: Into the Spider Verse*) in the separate waiting area during the pre- and post-lecture testing.

**Figure 1.**
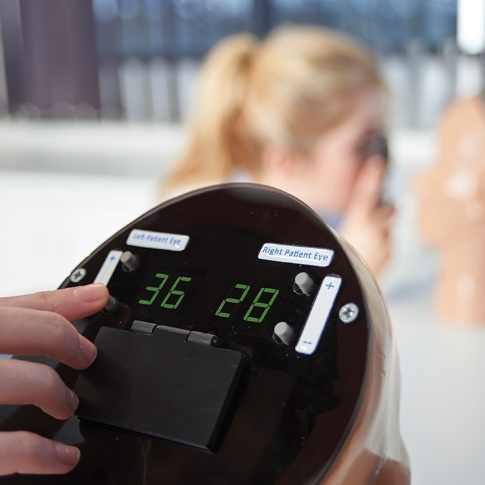
Adam Brouilly AR103 and AR403 Retinopathy Trainer. These are high resolution digital screen devices used to train providers in up to 36 different retinal conditions, requiring the use of an ophthalmoscope.

For the assessment of the emergency medicine residents’ funduscopic examination knowledge, we designed an image-based, 10-question exam with 15 possible answer choices (Appendix 1). More specifically, participants were shown 10 different pathologies in the simulation and for each they had to identify the correct pathology from a list of 15 choices. AR103B and AR403 digital eye examination retinopathy trainers were stationed in two separate examination rooms. These trainers’ devices allow for simultaneously deploying different high-resolution digital funduscopic and retinal condition images in the left and right eye by imputing a number into the rear of the trainer (Figure 1).

We randomized the 10-question exam for the individual eye on the trainer, resulting in four possible question and answer sheets, labeled as I-IV. Each of the two rooms had an assigned independent examiner responsible for inputting the numbers from the answer sheet and properly timing the study participants. For optimal efficiency and speed, each room would accommodate two study participants at a time, one assigned for the right eye and the other to the left. Each study participant would have 30 seconds to view the image in the designated eye and then an additional 30 seconds to select an answer from the answer sheet provided. While one study participant answered the question, the other would observe with the eye designated to them for that duration of 30 seconds. This alternating pattern between the two study participants would continue until all 10 questions had been answered, at which point two new study participants would be introduced to repeat the entire process.

The answer sheets in each room were labeled with a uniquely identifying letter designation at the top: A-L in room one, and M-Z in room two. Participants were not randomized into separate groups. Each participant served as their own control in a paired before and after design. As such, the letter assignment was only to ensure a participant’s pre- and post-training tests were able to be matched up for analysis, and for blinding the statistician from identifying participants while doing the scoring and analysis. Upon arrival to the digital retinopathy trainer, the study participants would be given two sheets bearing the same letter designation. On both answer sheets, the study participant would then write next to the letter designation their year of training, one through four, and whether they had completed an ophthalmology rotation prior to this study. On only one of the answer sheets, the independent examiner would write the Roman numeral indicating which simulated eye and pathology order are to be used. Once the aforementioned process of testing was completed, the pre-lecture answer sheets were collected and placed in a secure storage container. After completing the first round of testing, the study participant brought the second answer sheet to the waiting area.

In the waiting area, after all study participants had completed the initial round of pre- lecture testing, a concise lecture was presented on all 15 possible answer choices. The lecture used digital images obtained from the AR403 manual.

We proceeded with a second round of post-lecture testing. After the study participants completed their answer sheets following the initial examination, they were instructed to relocate to a different examination room, use a different digital eye examination trainer, and take a different Roman numeral test. The two study participants followed the same alternating pattern until they had completed all 10 questions of the post-lecture test. The study participants were not able to review the question-and-answer sheets after the study concluded. The post-lecture answer sheets were stored in a secure storage container.

### Statistical Analysis

The data was managed and preliminarily analyzed using Microsoft Excel (Microsoft® Excel® for Microsoft 365 MSO (Version 2308 Build 16.0.16731.20542) 32-bit, Microsoft Inc), with the Analysis Tool Pak Excel Add-In Package. Additional analyses were performed using R Statistical Software (R Version 3.3.2 [2016-10-31]). The small sample size of this study is a limitation to consider which may limit generalizability of the findings. However, this study utilized a sample of convenience based on the fixed number of emergency medicine residents in the residency program. The Shapiro-Wilk test was used to not reject the assumption of normality (p=0.162). A paired, 2-sided *t* test was used to determine if the mean change in test score was statistically significant. A 2-sample, 2-sided *t* test was used to check for differences by resident year and by ophthalmology rotation status within each round of the testing. Significance testing was performed at *P*<.05. All analyses were performed by David Metcalf, Research Statistician, at Henry Ford Jackson Health.

## RESULTS

To test the primary hypothesis that using a combination of lecture and simulation would increase the likelihood of accurate fundoscopic pathologies, the same 20 participants took both the before training test (control, n=20) and the after-training test (intervention, n = 20) groups. There were no missing data. Secondary analyses compared groups based on resident year and ophthalmology rotation status. Table 1 displays the number of participants in each subgroup.

**Table 1.** Case counts by subgroup

	Residency Year		Ophthalmology Rotation Completed	
	1^st^ or 2^nd^ year	3^rd^ or 4^th^ year	Yes	No
Pre-Test	12	8	11	9
Post-Test	12	8	11	9

**Figure 2.**
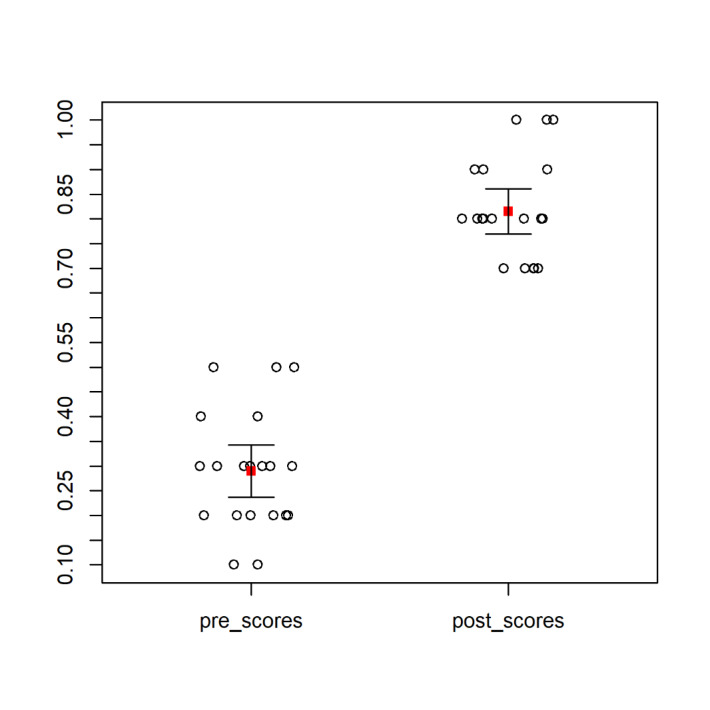
Graph of Pre-Test and Post-Test Score Means (red square). Including 95% CI (boxplot extensions out from the mean). The absence of intersection suggests a statistical significance.

**Figure 3.**
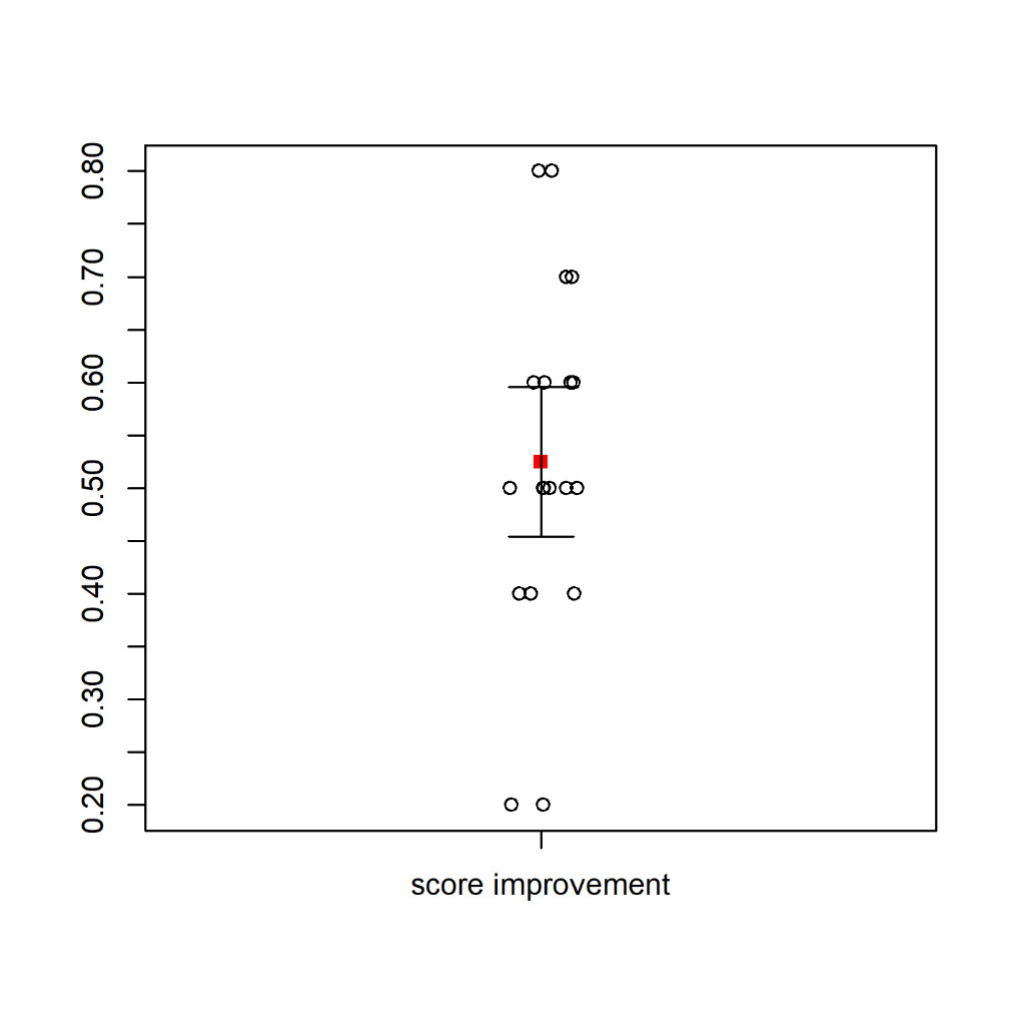
Graph of difference in Means between the Pre-Test and Post-Test Scores (red square). Including 95% CI (box plot extensions out from the mean). Since the 95% CI does not cross zero, this is a visual indication of significance.

All test scores were recorded and analyzed on the standard 0% - 100% scale. As anticipated, the mean test score improvement was quite large at 52.5% (SD = 16.2), 95% CI [44.9, 60.1], p=0.001. (Figure 2 and Figure 3)

When comparing the 3rd or 4th year residents to those in their 1st or 2nd year, the less experienced residents scored marginally better in the pre-test scoring with a mean difference of 2.5% (SED = 5.6), 95% CI [-9.4, 14.4], p=0.663. For the post test score, the more senior residents scored marginally better with a mean difference of 3.8% (SED = 4.8), 95% CI [-6.3, 13.8], p=0.444.

When comparing residents who had completed their ophthalmology rotation against those who had not, the ophthalmology rotation group scored marginally better in the pre-test scoring with a mean difference of 0.2% (SED = 5.6), 95% CI [-11.5, 11.9], p=0.972. For the post-test score, those who had completed their ophthalmology rotation scored noticeably higher than their non-ophthalmology counterparts with a mean score difference of 4.7% (SED = 4.7), 95% CI [-5.1, 14.6, p=0.323.

## DISCUSSION

Simulation-based training is now an integral part of medical training in the developed world. There is an increasing trend to put emphasis on simulation-based encounters over clinical encounters due to ethical considerations for the patient.^10^ In rare disease processes, there is also difficulty in finding appropriate patients who show clinical exam findings of interest. This raises the question of reproducibility in an actual clinical examination, which is beyond the scope of this study.

**Figure 4.**
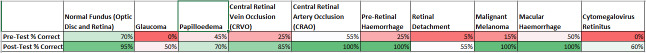
Additional pathology level pre-post improvements.

To reap any potential benefit from simulation-based training, correct diagnosis must be improved in the simulated clinical environment. The primary hypothesis was that we would see an improvement in participant’s scores after supplemental education. Indeed, the mean test score improved dramatically after the supplemental education via lecture, mean improvement of 52.5% (SD = 16.2%), 95% CI [44.9, 60.1], p=0.001. (29.0% [12.1] vs 81.5% [10.4], P <0.001). Although this may be secondary to other factors such as the participants being more familiar with the test equipment, the more likely interpretation is that correct diagnosis of the simulated retina is possible with recent supplemental education. Papilloedema was correctly diagnosed 45% pre-lecture and 70% post-lecture. Central Retinal Vein Occlusion increased from 25% pre to 85% post-lecture, and Malignant Melanoma increased from 15% pre-lecture to 85% post lecture. The need for emergent consultation vs urgent follow-up vs routine follow up may vary by clinical history, symptomatology, and other factors, but most of the pathologies for this pilot study were chosen due to their high acuity and “can’t miss” status for emergency medicine physicians. See figure 4 for additional pathology level pre-post improvements.

Both senior resident status and completing an ophthalmology rotation did not show a statistically significant difference in the scores of pre and post supplement education testing. This finding is interesting but not particularly surprising in view of the previously mentioned decline in funduscopic examination training and the rarity of some of these clinical pathologies. Also, the study had a small sample size due to the restricted pool of prospective clinical participants, which presented a major limitation that constrained proper extrapolation from the obtained data. To increase the sample size for a potential follow-up study, this intervention could be performed on all Emergency Medicine residents in the Henry Ford System. Although this simulation-based intervention could be extended to other specialties, however, the vast majority of selected clinical images were deemed most suitable for requiring an emergent follow-up with ophthalmology and may be less useful for or applicable to other specialties.

Of interest, the clinical condition most correctly identified on the pre-supplemental education examination was normal fundus (14 out of 20 respondents), and all but one respondent correctly identified the normal fundus after supplemental education. Also, four conditions showed 100% recognition after the supplement education: Central Retinal Artery Occlusion (CRAO), Pre-Retinal Hemorrhage, Malignant Melanoma, and Macular Hemorrhage. The bright spot, no pun intended, shows that at least most respondents know what normal looks like and can quickly obtain an accurate diagnosis in simulated training after minimal supplemental education.

A potential future research goal would be to repeat the ophthalmological examination test in the same cohort in three months to see the rate of retention. A repetitive training regimen with the AR403 would theoretically make this cohort of residents more likely to correctly identify pathology. The more difficult task of translating the success of simulated training to actual clinical training remains to be seen. Direct ophthalmoscopy remains an important part of an emergency physician’s diagnostic toolkit. More research needs to be done on the best modality for teaching this vital skill to residents, or else we face losing it.

### Conflict of Interest

None

## APPENDIX 1

**Table:**

	Question 1
	Normal Fundus
	Diabetic Retinopathy
	Malignant Melanoma
	Central Retinal Vein Occlusion
	Central Retinal Artery Occlusion
	Branch Retinal Vein Occlusion
	Pre Retinal Hemorrhage
	Multiple Retinal Hemorrhages
	Drusen
	Macular Hemorrhage
	Cytomegalovirus Retinitis
	Glaucoma
	Retinal Detachment
	Hypertensive Retinopathy
	Papilledema

**Table:**

	Question 2
	Normal Fundus
	Diabetic Retinopathy
	Malignant Melanoma
	Central Retinal Vein Occlusion
	Central Retinal Artery Occlusion
	Branch Retinal Vein Occlusion
	Pre Retinal Hemorrhage
	Multiple Retinal Hemorrhages
	Drusen
	Macular Hemorrhage
	Cytomegalovirus Retinitis
	Glaucoma
	Retinal Detachment
	Hypertensive Retinopathy
	Papilledema

**Table:**

	Question 3
	Normal Fundus
	Diabetic Retinopathy
	Malignant Melanoma
	Central Retinal Vein Occlusion
	Central Retinal Artery Occlusion
	Branch Retinal Vein Occlusion
	Pre Retinal Hemorrhage
	Multiple Retinal Hemorrhages
	Drusen
	Macular Hemorrhage
	Cytomegalovirus Retinitis
	Glaucoma
	Retinal Detachment
	Hypertensive Retinopathy
	Papilledema

**Table:**

	Question 4
	Normal Fundus
	Diabetic Retinopathy
	Malignant Melanoma
	Central Retinal Vein Occlusion
	Central Retinal Artery Occlusion
	Branch Retinal Vein Occlusion
	Pre Retinal Hemorrhage
	Multiple Retinal Hemorrhages
	Drusen
	Macular Hemorrhage
	Cytomegalovirus Retinitis
	Glaucoma
	Retinal Detachment
	Hypertensive Retinopathy
	Papilledema

**Table:**

	Question 5
	Normal Fundus
	Diabetic Retinopathy
	Malignant Melanoma
	Central Retinal Vein Occlusion
	Central Retinal Artery Occlusion
	Branch Retinal Vein Occlusion
	Pre Retinal Hemorrhage
	Multiple Retinal Hemorrhages
	Drusen
	Macular Hemorrhage
	Cytomegalovirus Retinitis
	Glaucoma
	Retinal Detachment
	Hypertensive Retinopathy
	Papilledema

**Table:**

	Question 6
	Normal Fundus
	Diabetic Retinopathy
	Malignant Melanoma
	Central Retinal Vein Occlusion
	Central Retinal Artery Occlusion
	Branch Retinal Vein Occlusion
	Pre Retinal Hemorrhage
	Multiple Retinal Hemorrhages
	Drusen
	Macular Hemorrhage
	Cytomegalovirus Retinitis
	Glaucoma
	Retinal Detachment
	Hypertensive Retinopathy
	Papilledema

**Table:**

	Question 7
	Normal Fundus
	Diabetic Retinopathy
	Malignant Melanoma
	Central Retinal Vein Occlusion
	Central Retinal Artery Occlusion
	Branch Retinal Vein Occlusion
	Pre Retinal Hemorrhage
	Multiple Retinal Hemorrhages
	Drusen
	Macular Hemorrhage
	Cytomegalovirus Retinitis
	Glaucoma
	Retinal Detachment
	Hypertensive Retinopathy
	Papilledema

**Table:**

	Question 8
	Normal Fundus
	Diabetic Retinopathy
	Malignant Melanoma
	Central Retinal Vein Occlusion
	Central Retinal Artery Occlusion
	Branch Retinal Vein Occlusion
	Pre Retinal Hemorrhage
	Multiple Retinal Hemorrhages
	Drusen
	Macular Hemorrhage
	Cytomegalovirus Retinitis
	Glaucoma
	Retinal Detachment
	Hypertensive Retinopathy
	Papilledema

**Table:**

	Question 9
	Normal Fundus
	Diabetic Retinopathy
	Malignant Melanoma
	Central Retinal Vein Occlusion
	Central Retinal Artery Occlusion
	Branch Retinal Vein Occlusion
	Pre Retinal Hemorrhage
	Multiple Retinal Hemorrhages
	Drusen
	Macular Hemorrhage
	Cytomegalovirus Retinitis
	Glaucoma
	Retinal Detachment
	Hypertensive Retinopathy
	Papilledema

**Table:**

	Question 10
	Normal Fundus
	Diabetic Retinopathy
	Malignant Melanoma
	Central Retinal Vein Occlusion
	Central Retinal Artery Occlusion
	Branch Retinal Vein Occlusion
	Pre Retinal Hemorrhage
	Multiple Retinal Hemorrhages
	Drusen
	Macular Hemorrhage
	Cytomegalovirus Retinitis
	Glaucoma
	Retinal Detachment
	Hypertensive Retinopathy
	Papilledema
